# High disparity in repellent gland anatomy across major lineages of stick and leaf insects (Insecta: Phasmatodea)

**DOI:** 10.1186/s40850-023-00189-2

**Published:** 2024-01-02

**Authors:** Marco Niekampf, Paul Meyer, Felix S. C. Quade, Alexander R. Schmidt, Tim Salditt, Sven Bradler

**Affiliations:** 1 Department of Animal Evolution and Biodiversity, Johann-Friedrich-Blumenbach Institute of Zoology and Anthropology, University of Göttingen, Untere Karspüle 2, 37073 Göttingen, Germany; 2 Institute for X-Ray Physics, University of Göttingen, Friedrich-Hund-Platz 1, 37077 Göttingen, Germany; 3 Department of Developmental Biology, Göttingen Center for Molecular Biosciences, Johann-Friedrich-Blumenbach Institute of Zoology and Anthropology, University of Göttingen, Justus-Von-Liebig-Weg 11, 37077 Göttingen, Germany; 4 Present address, Institut Für Zelltechnologie, Blücherstraße 63, 18055 Rostock, Germany; 5 Department of Geobiology, University of Göttingen, Goldschmidtstraße 3, 37077 Göttingen, Germany

**Keywords:** Comparative morphology, Defensive strategy, Insect evolution, Repellent glands, Secondary defense

## Abstract

**Background:**

Phasmatodea are well known for their ability to disguise themselves by mimicking twigs, leaves, or bark, and are therefore commonly referred to as stick and leaf insects. In addition to this and other defensive strategies, many phasmatodean species use paired prothoracic repellent glands to release defensive chemicals when disturbed by predators or parasites. These glands are considered as an autapomorphic trait of the Phasmatodea. However, detailed knowledge of the gland anatomy and chemical compounds is scarce and only a few species were studied until now. We investigated the repellent glands for a global sampling of stick and leaf insects that represents all major phasmatodean lineages morphologically via µCT scans and analyzed the anatomical traits in a phylogenetic context.

**Results:**

All twelve investigated species possess prothoracic repellent glands that we classify into four distinct gland types. 1: lobe-like glands, 2: sac-like glands without ejaculatory duct, 3: sac-like glands with ejaculatory duct and 4: tube-like glands. Lobe-like glands are exclusively present in *Timema*, sac-like glands without ejaculatory duct are only found in *Orthomeria*, whereas the other two types are distributed across all other taxa (= Neophasmatodea). The relative size differences of these glands vary significantly between species, with some glands not exceeding in length the anterior quarter of the prothorax, and other glands extending to the end of the metathorax.

**Conclusions:**

We could not detect any strong correlation between aposematic or cryptic coloration of the examined phasmatodeans and gland type or size. We hypothesize that a comparatively small gland was present in the last common ancestor of Phasmatodea and Euphasmatodea, and that the gland volume increased independently in subordinate lineages of the Occidophasmata and Oriophasmata. Alternatively, the stem species of Neophasmatodea already developed large glands that were reduced in size several times independently. In any case, our results indicate a convergent evolution of the gland types, which was probably closely linked to properties of the chemical components and different predator selection pressures. Our study is the first showing the great anatomical variability of repellent glands in stick and leaf insects.

**Supplementary Information:**

The online version contains supplementary material available at 10.1186/s40850-023-00189-2.

## Background

**Fig. 1:**
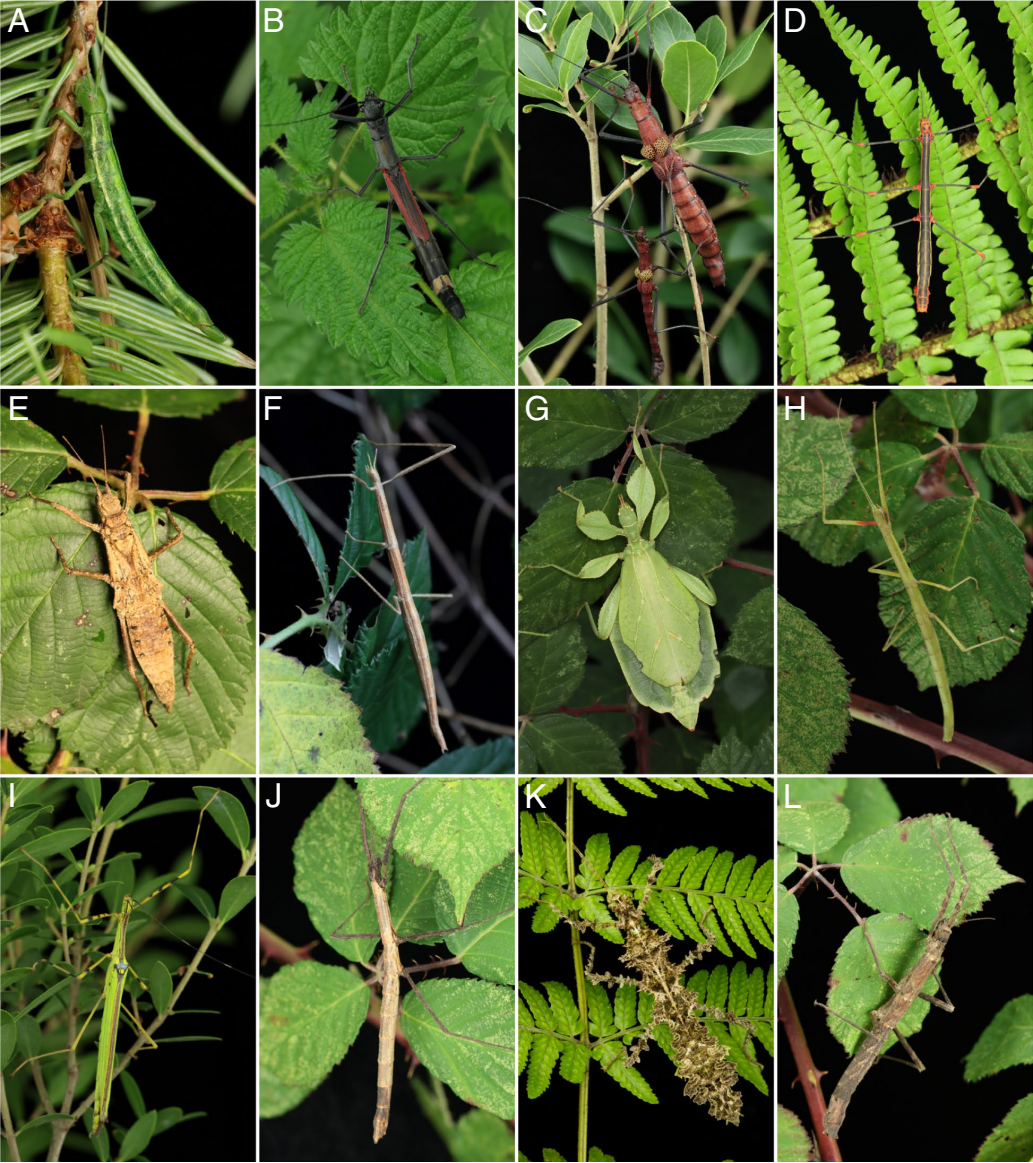
Overview of the phasmatodean species examined in this study. Females or couples (male individual always smaller) of **A**: *Timema douglasi*, **B**: *Orthomeria kangi*, **C**: *Pseudophasma subapterum*, **D**: *Oreophoetes peruana*, **E**: *Tisamenus fratercula*, **F**: *Clonopsis gallica*, **G**: *Phyllium philippinicum*, **H**: *Carausius morosus*, **I**: *Anarchodes annulipes*, **J**: *Lobofemora scheirei*, **K**: *Taraxippus samarae*, **L**: *Dimorphodes* sp

Predation constitutes an ultimate selective pressure on animal morphology, physiology, and behavior, with immediate and irrevocable fitness consequences for ineffective strategies [1–4]. Thus, predator–prey interactions are an important driving force in evolution. An optimal antipredator strategy may involve multiple traits and various behaviors performed simultaneously or sequentially [5]. Stick and leaf insects, traditionally referred to as insect order Phasmatodea, are well known for their astonishing camouflage capabilities, exhibiting extreme forms of masquerade crypsis or plant mimicry whereby they phenotypically resemble twigs (Fig. 1F, H), bark (Fig. 1E, J, L), lichens or mosses (Fig. 1K), and live (green) (Fig. 1G) or dead (brown) leaves [6, 7]. Anatomical characteristics such as an extremely elongated or leaf-like expanded body enable these predominantly nocturnal insects to remain undetected by predators (crypsis) or being misidentified as inanimate objects (masquerade) [8]. The insects usually remain motionless during the daytime (catalepsy), but display a swaying behavior when blown by wind, thus resembling wind-blown vegetation, a phenomenon referred to as motion camouflage [9]. These successful primary defensive strategies, i.e., those effective in absence of any predator and thus favoring detection avoidance, are assisted by a wide range of secondary defensive strategies, i.e., those effective after detection and attack by a predator, which involves flight, thanatosis, defensive stridulation, counterattack and threatening gestures like startle display or deimatic behavior, in which the insects attempt to intimidate the attacker by presenting a certain physical defensiveness or by the sudden revelation of warning colors from previously hidden body parts [10, 11]. Additionally, stick and leaf insects are capable to defend themselves chemically with defensive secretions [6, 11].

Chemical defense is a widely used strategy to repel attackers in a variety of insect taxa, such as Dermaptera, Orthoptera, Blattodea, Hemiptera, Hymenoptera, Neuroptera and Coleoptera, and repellent secretions are applied through a wide range of morphological features [12]. In Phasmatodea, the application of defensive substances is particularly well developed in conspicuous species with aposematic coloration (Fig. 1C, I) indicating inedibility such as the Peruvian fire stick *Oreophoetes peruana* (Saussure, 1868) (Fig. 1D) and the southern two-striped walkingstick, or devil rider, *Anisomorpha buprestoides* (Houttuyn, 1813) from Florida [12]. The repellent substances are produced and emitted via repellent glands that are located pairwise in the thorax, adjacent to the digestive system with one dorsolaterally opening each at the anterior margin of the prothorax [13, 14]. The glands originate from invaginations of the outer cuticle, which is underlain basally by a single-layered glandular epithelium, where the defensive secretion is produced [13]. The defensive substances are released via contraction of musculature that surrounds the glandular epithelium. The tissue organization has been previously outlined by Eisner (1965), Happ et al. (1966), Strong (1975), van de Kamp et al. (2015) and Strauß et al. (2017) [13–17]. The presence of these glands is considered a derived autapomorphic trait of the Phasmatodea and, in consequence, these glands were assumed to be widely present among the approximately 3500 known species of stick and leaf insects [17–19]. However, only few studies were conducted on this defensive system, with few detailed descriptions available [13, 14, 17, 20–22] and only brief depiction or mention of this character system in species descriptions [23–27]. According to the sparse information available, the repellent glands vary significantly in size and exhibit a high degree of species-specific morphological disparity. The glands of *Anisomorpha* spp. females measure approximately 1 cm and reach up to the end of the elongated mesothorax [17, 28], whereas the glands of female *Sipyloidea sipylus* (Westwood, 1859) are merely 1.5 mm long, or do not exceed beyond the middle of the short prothorax [29]. Additionally, there are huge differences in the general glandular anatomy. In *Anisomorpha buprestoides* and *Peruphasma schultei* Conle & Hennemann, 2005, the glands are formed as uniform plain long tubes [16, 28]. In contrast, *Diapheromera femorata* (Say, 1824) and *Oreophoetes peruana* have significantly smaller glands, formed as sacs, with a thin duct leading to the glandular opening [20, 30]. The malodorous secretions that stick insects release to repel attackers [17–19] also show a high degree of chemical diversity [31]. To date, the chemical components of the repellent secretion of twelve phasmatodean species have been analyzed, and at least 27 different substances have been identified. The majority of the studied species produce monoterpenes, such as actinidine (*Megacrania tsudai* Shiraki, 1933) and peruphasmal (*Peruphasma schultei)*, while other species produce alkyldimethylpyrazines (*Cryptophyllium westwoodii* (Wood-Mason, 1875)) and quinoline (*Oreophoetes peruana*) [20, 32–34]. The majority of identified substances is highly irritating to the eyes and mucous membranes. They serve as effective repellents against predators and parasites such as spiders, ants, mosquitoes, beetles, parasitic wasps, mice, rats, frogs, lizards and birds, and can be either directly sprayed towards attackers or spread on the insect’s body surface [15, 20, 29, 35–37].

Although Scudder (1876) first described the repellent glands of stick insects already more than 140 years ago, knowledge in this regard is very scarce, with only few details for individual species reported, whereas a broad comparative approach is missing.

Here, we describe and compare the anatomy of repellent glands of twelve species representing all major phasmatodean lineages via micro-computed tomography (µCT). µCT with micro- and nano-focus X-ray sources has opened three-dimensional (3D) non-destructive imaging of the internal anatomy of small organisms with affordable laboratory instruments, at scalable resolution and field of view, where iodine staining and critical point drying provided particular advantages for tissue differentiation, in combination with automatic or semiautomatic segmentation [38]. Different preparation and staining techniques have been proposed to maximize contrast and tissue differentiation [39]. Furthermore, it is now well established that reconstruction schemes which exploit phase contrast can yield enhanced image quality for the anatomy of small organisms [40, 41].

Using µCT data, we show the characteristics and morphological disparity across the phylogeny of stick and leaf insects. Except for *Oreophoetes peruana*, none of the species has been the subject of previous repellent gland related studies. Our approach serves as a first step towards understanding the evolution of this vital but previously neglected character system. We propose a classification of the repellent glands into four distinct types, 1: lobe-like glands, 2: sac-like glands without ejaculatory duct, 3: sac-like glands with ejaculatory duct, and 4: tube-like glands, and discuss their distribution across the Phasmatodea.

## Results

**Fig. 2:**
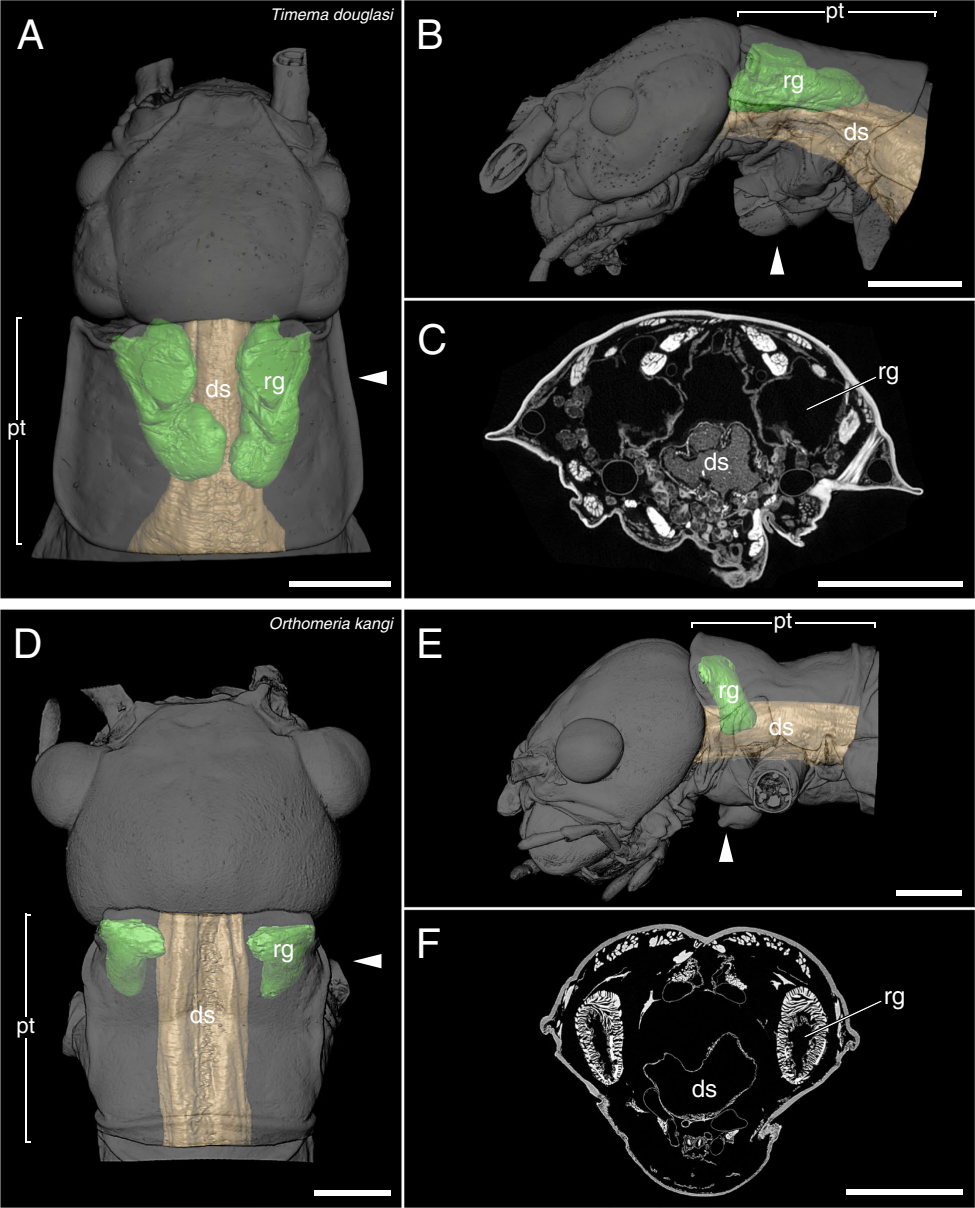
3D visualization and µCT scan cross section of lobe-like glands in *Timema douglasi* (**A**–**C**) and sac-like glands without ejaculatory duct in *Orthomeria kangi* (**D**–**F**). A&D: dorsal view, B&E: lateral view, C&F: µCT scan. ds = digestive system, pt = prothorax, rg = repellent gland, arrowhead = area of µCT scan arrowhead = level of cross-section of prothorax. Scale bars: 1 mm

**Fig. 3:**
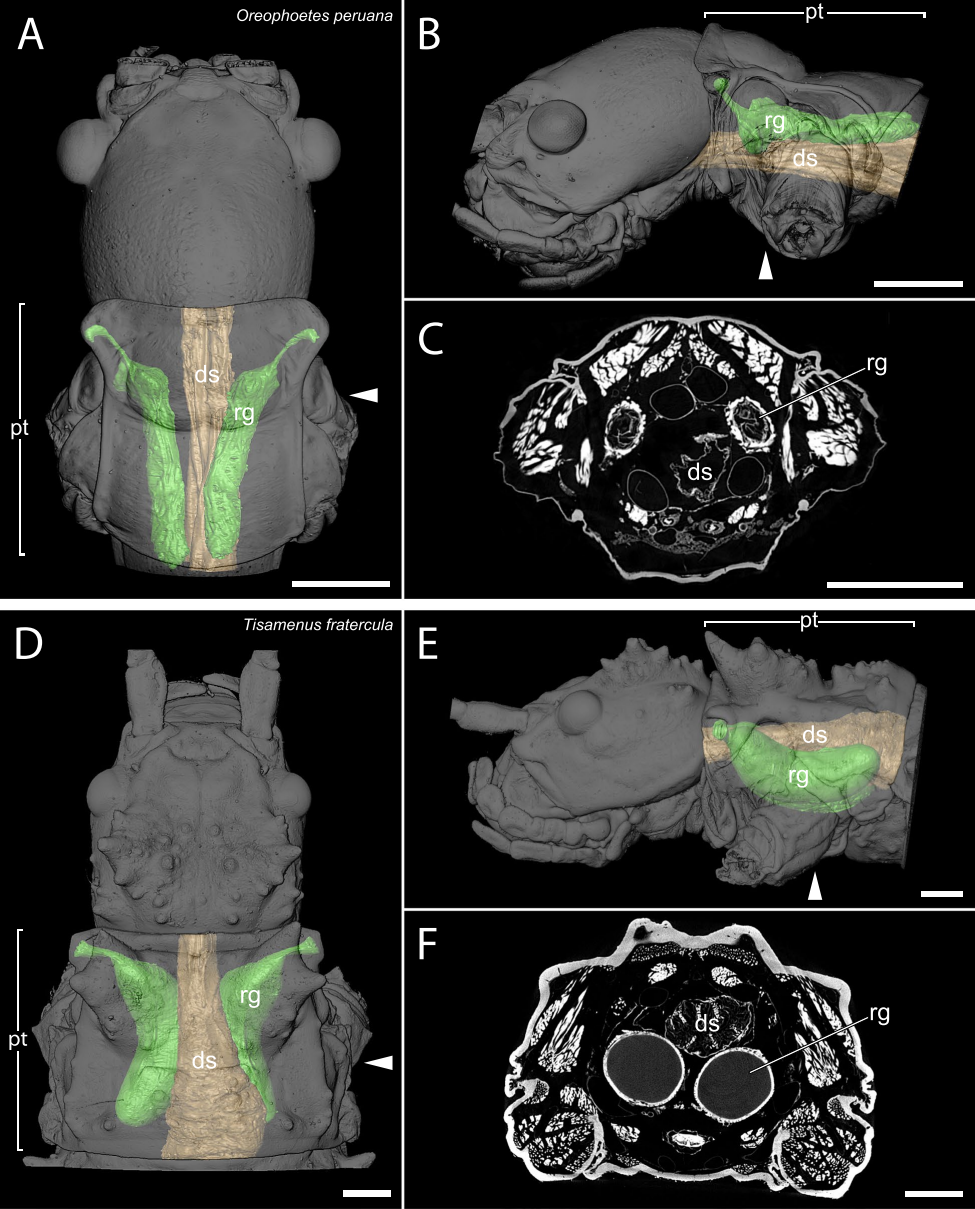
3D visualization and µCT scan cross section of sac-like glands with ejaculatory duct in *Oreophoetes peruana* (**A**–**C**) and *Tisamenus fratercula* (**D**–**F**). A&D: dorsal view, B&E: lateral view, C&F: µCT scan. Abbreviations as in Fig. 2. Scale bars: A–C = 1 mm, D–E = 2 mm

**Fig. 4:**
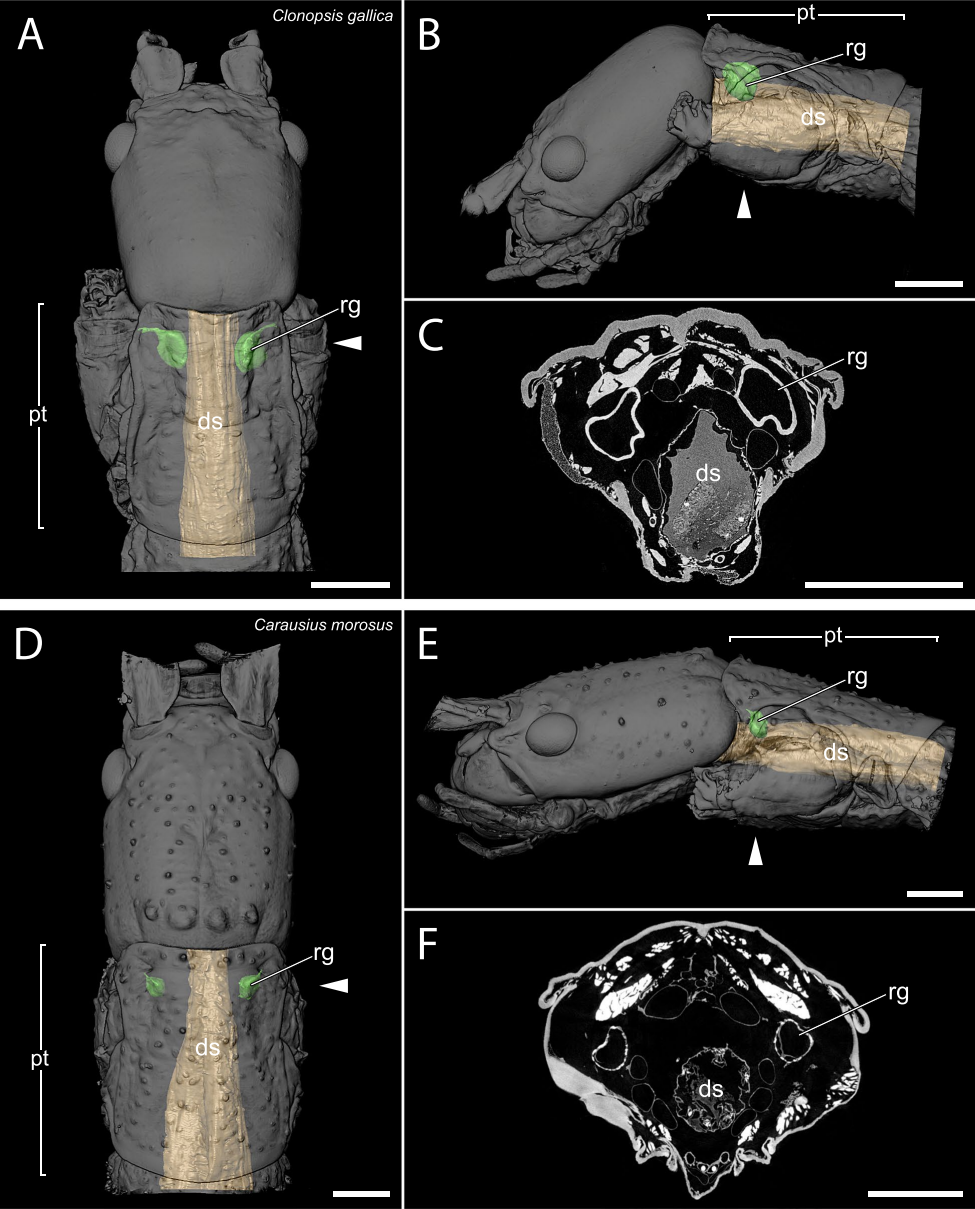
3D visualization and µCT scan cross section of sac-like glands with ejaculatory duct in *Clonopsis gallica* (**A**–**C**) and *Carausius morosus* (**D**–**F**). A&D: dorsal view, B&E: lateral view, C&F: µCT scan. Abbreviations as in Fig. 2. Scale bars: 1 mm

**Fig. 5:**
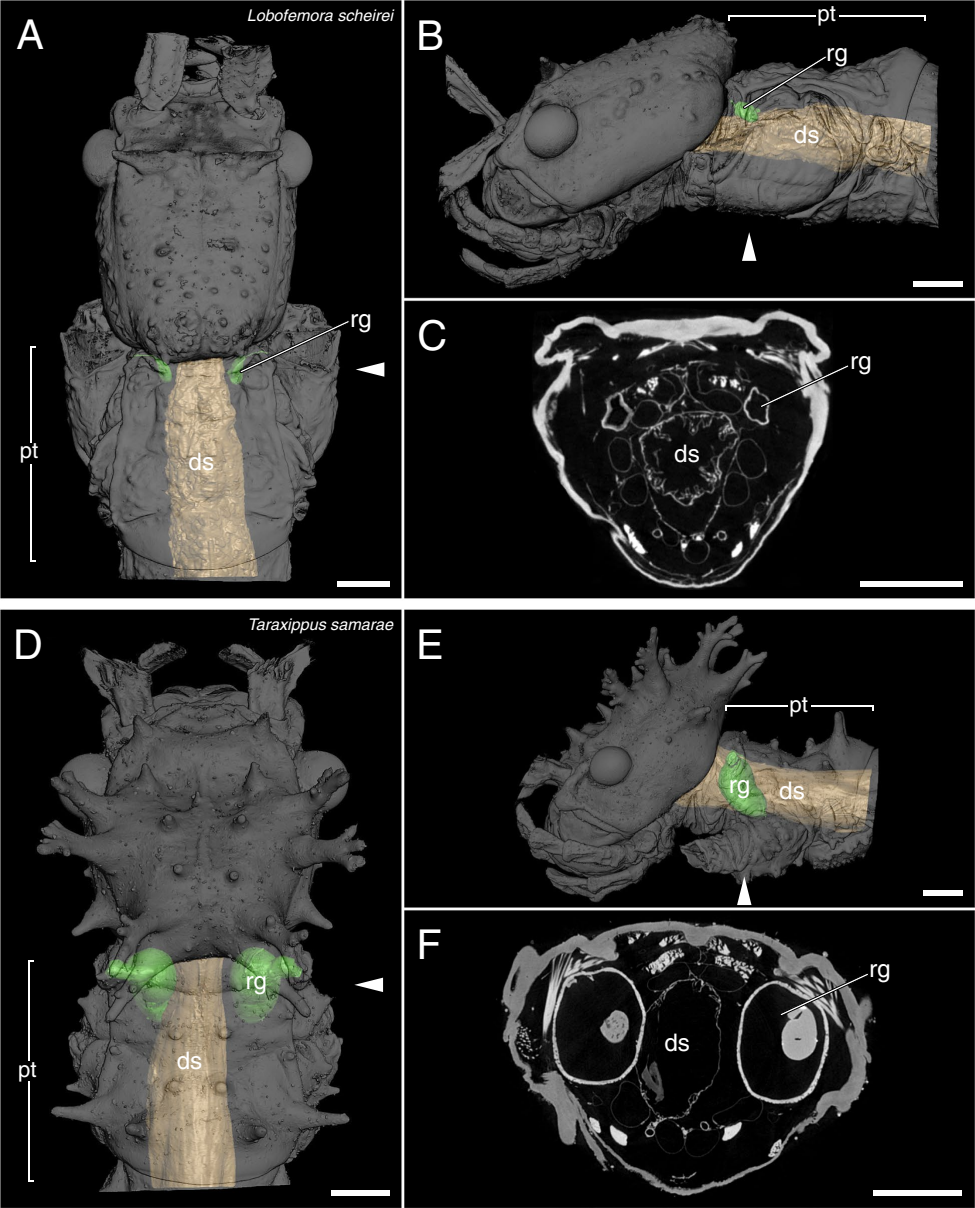
3D visualization and µCT scan cross section of sac-like glands with ejaculatory duct *Lobofemora scheirei* (**A**–**C**) and *Taraxippus samarae* (**D**–**F**). A&D: dorsal view, B&E: lateral view, C&F: µCT scan. Abbreviations as in Fig. 2. Scale bars: 1 mm

**Fig. 6:**
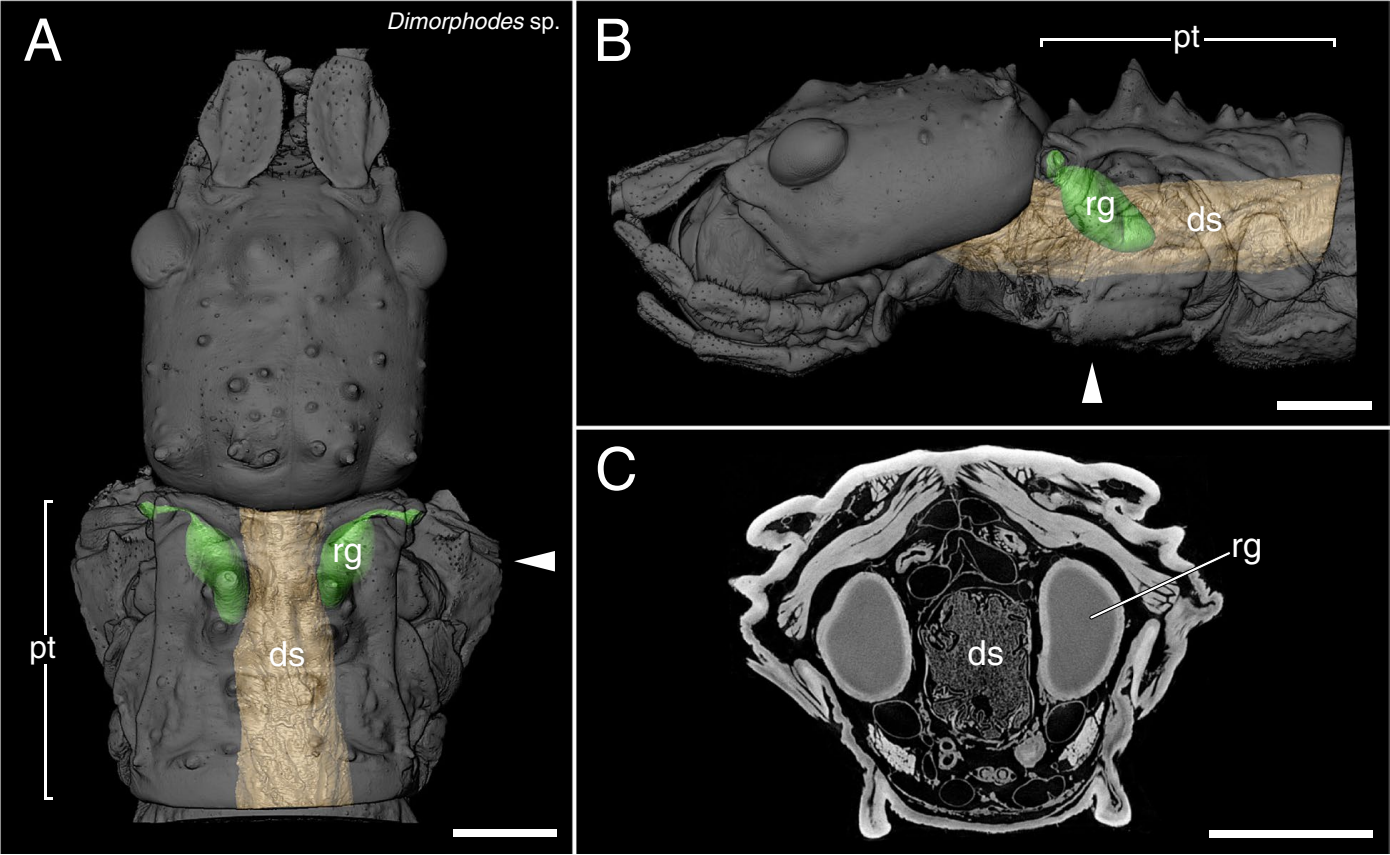
3D visualization and µCT scan cross section of sac-like glands with ejaculatory duct in *Dimorphodes* sp. **A**: dorsal view, **B**: lateral view, **C**: µCT scan. Abbreviations as in Fig. 2. Scale bars: 2 mm

**Fig. 7:**
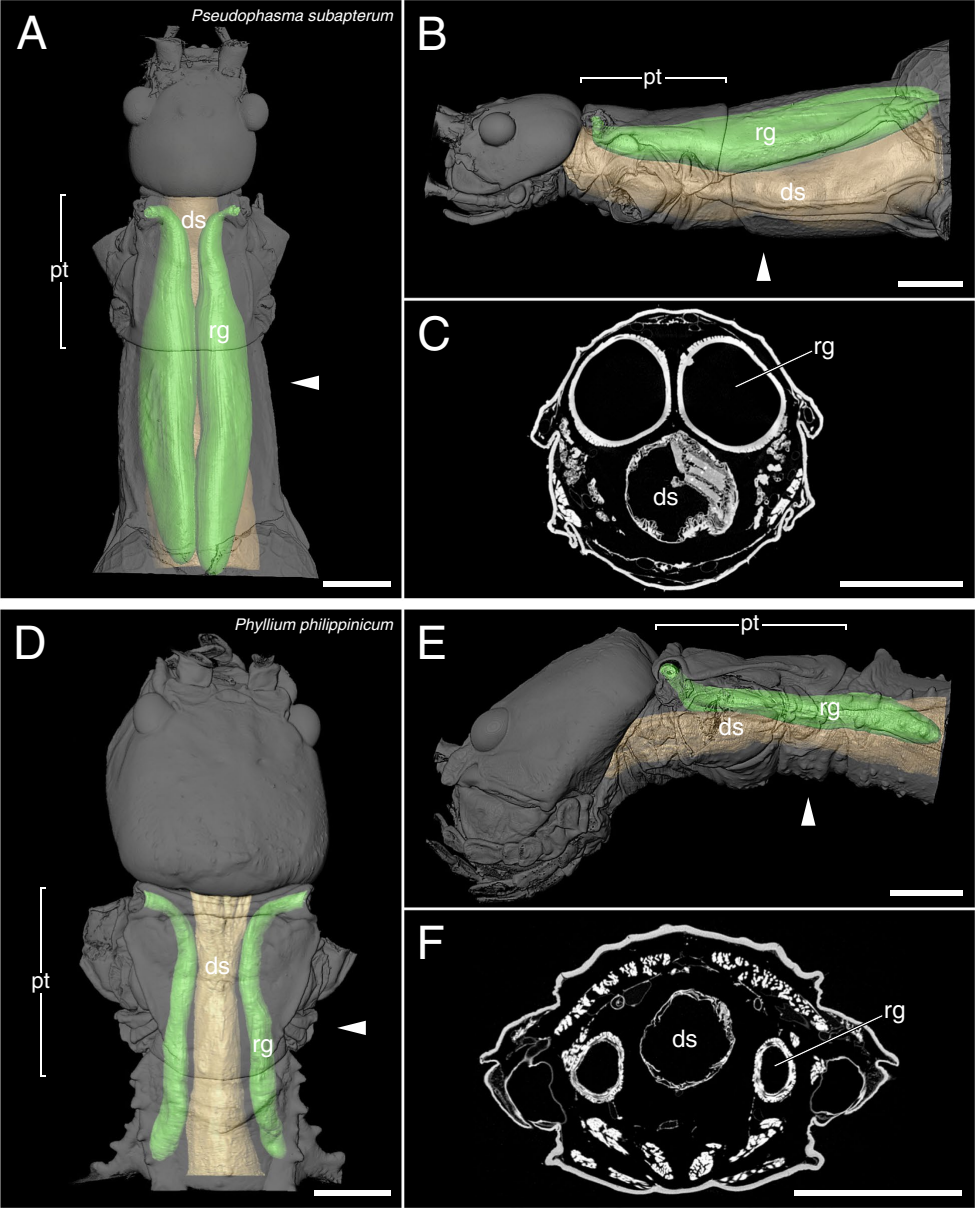
3D visualization and µCT scan cross section of tube-like glands in *Pseudophasma subapterum* (**A**–**D**) and *Phyllium philippinicum* (**D**–**F**): A&D: dorsal view, B&E: lateral view, C&F: µCT scan. Abbreviations as in Fig. 2. Scale bars: 2 mm

**Fig. 8:**
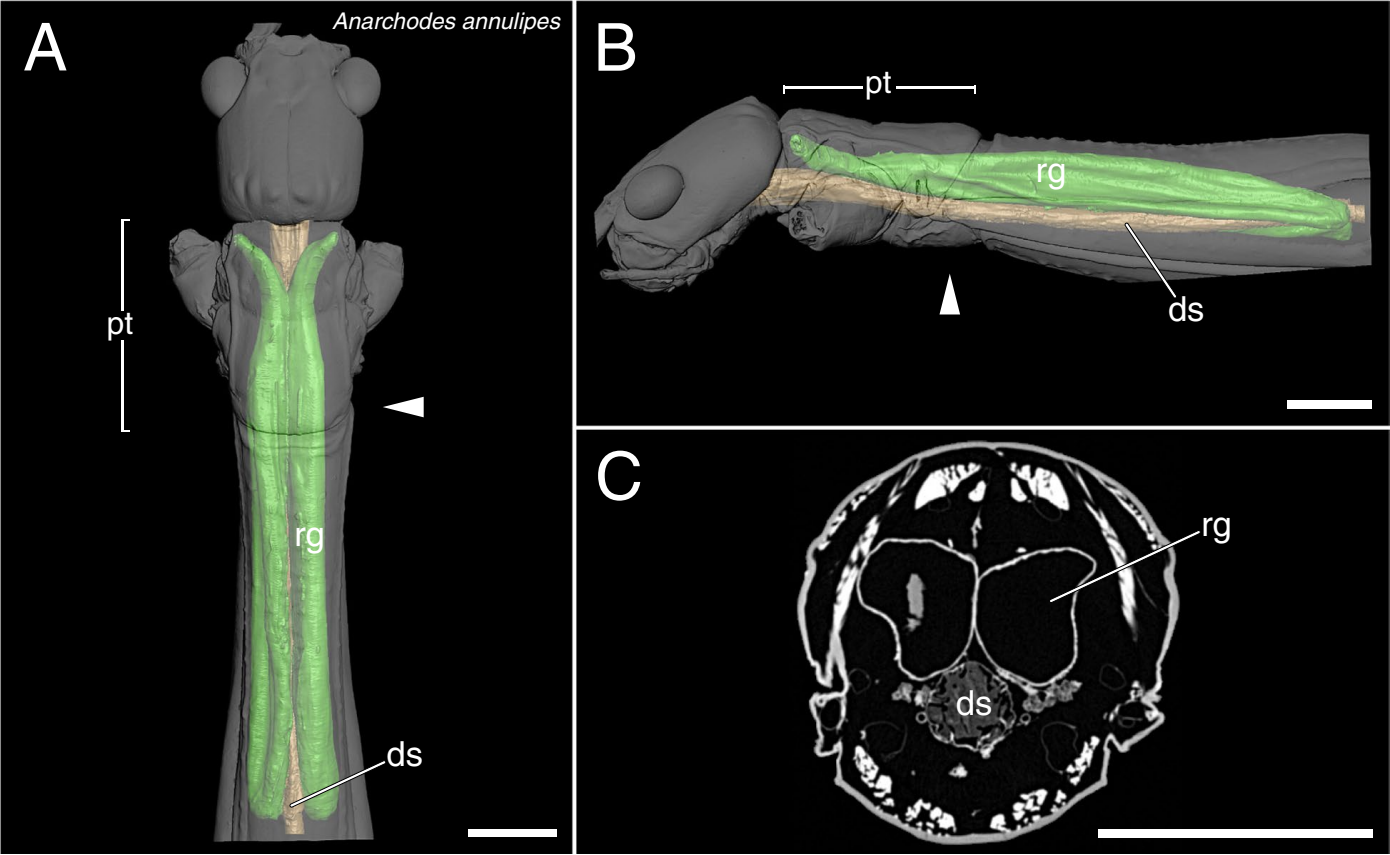
3D visualization and µCT scan cross section of tube-like glands in *Anarchodes annulipes*. **A**: dorsal view, **B**: lateral view, **C**: µCT scan. Abbreviations as in Fig. 2. Scale bars: 2 mm

**Fig. 9:**
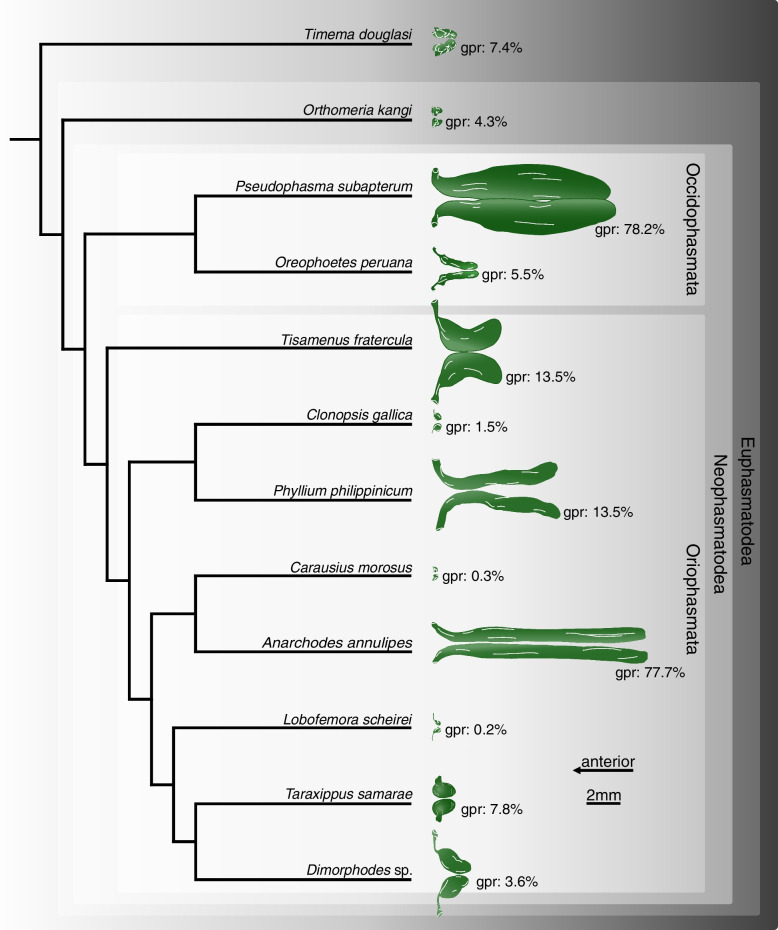
Repellent gland types in their absolute size mapped onto the phylogeny of the examined species based on Simon et al. 2019 [7] (dorsal view, to scale). Lobe-like glands in *Timema douglasi*; sac-like glands without ejaculatory duct in *Orthomeria kangi*; sac-like glands with ejaculatory duct in *Oreophoetes peruana*, *Tisamenus fratercula*, *Clonopsis gallica*, *Carausius morosus*, *Lobofemora scheirei*, *Taraxippus samarae*, *Dimorphodes* sp.; tube-like glands in *Pseudophasma subapterum*, *Phyllium philippinicum*, *Anarchodes annulipes*

The µCT scans revealed a high disparity of the prothoracic repellent glands in regard of size and structure (Figs. 2,3,4,5,6,7,8 and 9, Table 1). The absolute gland volume (left and right gland combined) ranges from 0.04 mm^3^ (*Lobofemora scheirei*) to 27.65 mm^3^ (*Pseudophasma subapterum*). The relative gland size (gland-prothorax ratio) lies between 0.2% (*Lobofemora scheirei*) and 78.2% (*Pseudophasma subapterum*), and the lumen-gland ratio ranges from 17% (*Orthomeria kangi*) to 88% (*Timema douglasi)*.

**Table 1:** Volume measurements in mm^3^, gland-prothorax ratios and lumen-gland ratios of the investigated species. Values rounded.

	Left gland volume	Right gland volume	Left lumen volume	Right lumen volume	Prothorax volume	Gland-prothorax ratio	Lumen-gland ratio
*Timema douglasi*	0.34	0.32	0.30	0.28	8.84	7.4%	88%
*Orthomeria kangi*	0.59	0.61	0.10	0.10	27.80	4.3%	17%
*Pseudophasma subapterum*	13.66	13.99	9.90	9.65	35.36	78.2%	71%
*Oreophoetes peruana*	0.28	0.27	0.14	0.14	9.92	5.5%	52%
*Tisamenus fratercula*	5.21	5.17	4.12	4.02	75.61	13.7%	78%
*Clonopsis gallica*	0.04	0.04	0.02	0.02	5.50	1.5%	57%
*Phyllium philippinicum*	2.86	2.56	1.38	1.32	40.07	13.5%	50%
*Carausius morosus*	0.03	0.02	0.02	0.01	14.88	0.3%	59%
*Anarchodes annulipes*	7.41	6.18	6.23	4.35	17.49	77.7%	78%
*Lobofemora scheirei*	0.02	0.02	0.01	0.01	18.17	0.2%	60%
*Taraxippus samarae*	0.83	0.93	0.68	0.74	22.53	7.8%	80%
*Dimorphodes* sp.	1.47	1.48	0.88	0.91	83.01	3.6%	61%

Within the twelve investigated species, we were able to distinguish between four principally different gland types, which we describe as 1: lobe-like glands, 2: sac-like glands without ejaculatory duct, 3: sac-like glands with ejaculatory duct and 4: tube-like glands.

1. Lobe-like glands

This gland type was exclusively present in *Timema douglasi* (Fig. 2A–C). The glands are relatively small and posteriorly reach to the middle of the prothorax. With a gland-prothorax ratio of only 7.4%, they exhibit a highly folded and wrinkled structure, being distinctly curved towards the digestive system, with a mesal lobe-like extension as described before in *Timema* [18]. The musculature and glandular epithelium are comparatively thin, and the glandular lumen constitutes a large part of the gland with a lumen-gland ratio of 88%.

(2) Sac-like glands without ejaculatory duct

Sac-like glands without ejaculatory duct were exclusively identified in *Orthomeria kangi* (Fig. 2D–F). These glands are likewise very small, with a gland-prothorax ratio of 4.3%, and do not exceed the anterior half of the prothorax. This type exhibits plain roundish structured glands without folding as in the lobe-like glands of *Timema*. The musculature is prominent in comparison and the lumen is relatively small with a lumen-gland ratio of 17%.

(3) Sac-like glands with ejaculatory duct

This prothoracic repellent gland type only differs from the sac-like glands in a few but important features. As in the previous type, the gland also exhibits a roundish sac-like structure, though strongly decreases in diameter towards the gland-opening forming an ejaculatory duct that is either surrounded by a distinctly thinner musculature (i.e., *Tisamenus fratercula, Taraxippus samarae*) or the musculature is missing (*O. peruana*). This type is the most abundant among the stick insects used in our study (present in seven species): *Oreophoetes peruana* (Fig. 3A-C), *Tisamenus fratercula* (Fig. 3D-F), *Clonopsis gallica* (Fig. 4A-C), *Carausius morosus* (Fig. 4D-F), *Lobofemora scheirei* (Fig. 5A-C), *Taraxippus samarae* (Fig. 5D-F), *Dimorphodes* sp. (Fig. 6A-C). The maximum gland-prothorax ratio exceeds the sac-like glands and ranges from 0.2% (*L. scheirei*) to 13.7% (*Ti. fratercula*). The relative muscle portion varies among species, with the lumen-gland ratio ranging from 52% (*O. peruana*) to 80% (*Ta. samarae*).

(4) Tube-like glands

The tube-like glands exceed the other gland types considerably both in absolute and relative size. This type was found in *Pseudophasma subapterum* (Fig. 7A–C), *Phyllium philippinicum* (Fig. 7D–F) and *Anarchodes annulipes* (Fig. 8A–C). The glands are developed as long tubes extending to the hind margin of the mesothorax. Their diameter slightly decreases anteriorly towards the gland opening, but not in the same way as in sac-like glands, thus not forming a distinct ejaculatory duct and a pronounced musculature is reaching up to the glandular opening. The gland-prothorax ratio ranges from 13.5% (*Ph. philippinicum*) to 78.2% (*Ps. subapterum*) and the lumen-gland ratio from 50% (*Ph. philippinicum*) to 78% (*A. annulipes*).

## Discussion

This is the first time µCT scans were applied to investigate the anatomy of the glands in Phasmatodea, which allowed differentiation between four morphologically distinct types. While type 1 (lobe-like glands) and type 2 (sac-like glands without ejaculatory duct) occur specifically in two early diverging lineages, *Timema* (Timematodea) and *Orthomeria* (Aschiphasmatidae) respectively, both types 3 (sac-like glands with ejaculatory duct) and 4 (tube-like glands) occur across all remaining stick insects or Neophasmatodea (Fig. 9). Hereby we could not detect any phylogenetic signal, i.e., species with the same type of defensive gland appear largely unrelated, whereas closely related taxa may exhibit fundamentally different gland types – and sizes, e.g., *Oreophoetes* and *Pseudophasma* in Occidophasmata, or *Clonopsis* and *Phyllium* in Oriophasmata (Fig. 9). It is apparent that the absolutely largest glands are generally tube-like glands (type 4: *Ps*. *subapterum*, *Ph*. *philippinicum*, *A*. *annulipes*), thus the ejaculatory duct might be dispensable above a certain gland size. However, when considering the relative gland size, i.e., the gland-prothorax ratio (gpr), two species with nearly identical gpr values in fact exhibit two different gland morphologies: *Ti*. *fratercula* (Heteropterygidae: Obriminae) with a gpr of 13.7% has a sac-like tube with ejaculatory duct (type 3), while *Ph*. *philippinicum* (Phylliidae) developed a tube-like gland (type 4) with a gpr of 13.8%. Alternatively, ejaculatory ducts could have evolved independently. Overall, the relative gland size differs enormously among phasmatodeans, with a gpr ranging from 0.2% in *L. scheirei* to 78.2% in *Ps. subapterum*, thus differing by a factor of nearly 400. Only one female individual per species was analyzed via µCT, so we did not infer any intraspecific variation of gland sizes. However, such variations could only slightly affect the gland volume measured, but not the principal gland types. The glands investigated were not emptied before dissection as specimens were extremely carefully processed. In the few cases where we observed partial spraying (obvious by observing size asymmetries and contracted areas in the gland pairs), we dismissed the individual from our study. Nonetheless, the gland volume must always be considered a minimum possible value, as we cannot ensure that the glands are entirely filled or whether larger glands might occur in a species. Yet, minor intraspecific differences in gland size would not affect the overall outcome of our study. The different gland types do not relate to evolutionary lineages, neither does gland size, with extremely large glands appearing in both Occidophasmata (e.g., *Pseudophasma*) and Oriophasmata (e.g., *Anarchodes*). Since the early evolutionary side branches *Timema* and *Orthomeria* possess small absolute and relative glands, we conclude that larger defensive glands did not appear before the last common ancestor of Neophasmatodea. However, based on our restricted taxon sampling we cannot perform a reliable ancestral character state analysis and cannot determine whether the stem species of Neophasmatodea already had large glands that were reduced multiple times in subordinate lineages or vice versa. In consequence, common ancestry does not appear to play a significant role in determination of the gland type and size.

We detected the presence of prothoracic repellent glands in all investigated stick and leaf insect taxa (for overview see Fig. 9). Tilgner (2001) stated in his cladistic analysis of phasmatodean relationship that all examined taxa possess prothoracic exocrine glands, but often the openings of the glands are not sclerotized, and the glands may thus appear to be absent unless a careful dissection is performed to reveal them [42]. However, Tilgner (2001) did not illustrate any repellent glands in his study but had described the gland of *Timema* c*ristinae* Vickery, 1993 [18] that largely corresponds to our finding in *Ti. douglasi*. In addition, our results are consistent with those of Stolz (2019) [22] concerning the repellent glands of *Ti. douglasi*. Since both taxa are distantly related within the genus [43], we can conclude that the described gland structure is likely uniform and representative for *Timema*. Only for one further taxon, the Peruvian fire stick *Oreophoetes*, previous anatomical studies are available [20, 28] that corroborate the gland reconstruction presented here. Moreover, the repellent glands of *Peruphasma schultei*, *Anisomorpha buprestoides* and *Anisomorpha paromalus* Westwood, 1859 are illustrated in several studies [16, 17, 28] and coincide in type and size with those of *Ps. subapterum* (Fig. 7A–C). Thus, we are confident that the tube-like glands are representative for the Pseudophasmatinae. For all remaining taxa we describe and illustrate the gland anatomy for the first time, although the presence of glands was mentioned before in some of them, i.e., *Carausius morosus* [44, 45].

It is crucial to decipher what alternative factors determine the glandular anatomy and what role the natural history and ecological factors play in this regard. Previous studies focused on prominent and conspicuous spraying phasmid species, e.g., the southern two-striped walkingstick *Anisomorpha buprestoides* [13, 15, 28] and the Peruvian fire Stick *Oreophoetes peruana* [20, 46], and thus gave the impression that aposematically colored species in particular have large defensive glands [47]. However, equally large glands and the same types of glands appear also to be present in non-aposematic and well camouflaged species. For instance, the bark mimic *Ti. fratercula* (Heteropteryginae, Fig. 1E) has the same gland type, but relatively and absolutely larger glands (cf. Figure 8) than the flamboyant *O. peruana* (Diapheromerinae, Fig. 1D). The leaf mimic *Ph. philippinicum* (Phylliidae; Fig. 1G) has much larger glands (Fig. 8) than the conspicuously colored species *O. kangi* (Aschiphasmatinae; Fig. 1B), albeit *Ps. subapterum* (Fig, 1C; Pseudophasmatidae) and *A. annulipes* (Fig. 1I; Necrosciinae), both strikingly colored species, have by far the biggest glands observed in our study, capturing more than 75% of the gland-prothorax ratio (Fig. 9).

Since the portion of the muscles and gland reservoir might vary significantly in relation to the whole gland (for overview see Table 1), this must also be taken into account when considering the total gland size. Glands of the same size might alternatively have large muscles surrounding a small reservoir (e.g., *O. kangi*, Fig. 2F) or small muscles surrounding a much larger reservoir (e.g., *Ta. samarae*, Fig. 5F). Since the thickness of the glandular epithelium, which in general is a single layer of cells [13], does not differ between species, the reservoir containing the repellent substance is mainly responsible for the size difference. We provided this information as the lumen-gland ratio (lgr) and observed all combinations with the gpr. A huge gpr with a huge lgr (e.g., *A. annulipes*) can be detected as well as a small gpr with a huge lgr (e.g., *Ta. samarae*) and a small gpr with a small lgr (e.g., *O. kangi*). However, we could not find species with a huge gpr and small lgr. The lgr value obviously describes the trade-off between more capacity for repellent secretion (bigger lumen) and a bigger musculature (smaller lumen) for more effective substance ejaculation. The glandular morphology, or gland type, affects the spraying mechanism. Secretions can be emitted in form of a spray, a volatile mist, a drop or a jet of liquid [28, 29, 46], with some species like *A. buprestoides* and *Megacrania batesii* Kirby, 1896 even being able to aim in different directions [15, 48]. However, there are species incapable of aiming: Even if attacked frontally at the head, individuals of *O. peruana* emit the secretion in a thin curved jet in posterior direction (pers. obs.). For different ways of ejection and aiming, different morphological adaptions are required, which can be deduced from the µCT scans. In sac-like glands with ejaculatory duct, the slender ducts may be helpful to build up a certain pressure, in order to emit the repellent substance over a certain distance, whereas in other gland types specific structures at the glandular opening serve the same purpose. Similar effects are described for the oral papillae of velvet worms (Onychophora) and the chelicerae of spitting spiders (Araneae: Scytodidae), where slender ducts are described to increase hydrostatic pressure and emitting speed [49, 50]. The slender ducts appear to have a further advantage for *O. peruana* as described by Eisner et al. (1997): The ducts are simply too narrow for the whole cuticular sac to be pulled out during moulting. Hence, the cuticular duct and sac, still containing the repellent substance remain inside the new gland reservoir. Thus, *O. peruana* is able to defend itself immediately after moulting, whereas other species that lose the whole gland and its content during the moult remain temporarily undefended until sufficient repellent substance is produced and restored [20, 51]. The remains of the smaller cuticular sacs of former stages are clearly visible inside the gland [20] and also visible in our µCT scans (Fig. 3C). However, this could not be confirmed for other species with similarly small ejaculatory ducts, where the old cuticular sac appears to be lost entirely during the moult. We conclude that this described mechanism of sustaining the chemical defensiveness during the vulnerable act of moulting is a specific adaptation that might have been crucial for the evolution of the aposematic coloration in *Oreophoetes*. In *Ti. fratercula* (Fig. 3F), *A. annulipes* (Fig. 8C), *Ta. samarae* (Fig. 5F) and *Dimorphodes* (Fig. 6C) other content can be observed inside the gland that does not represent remains of the cuticular sac. The glands were fixated in their filled state during the preparation process, and the repellent secretion is replaced in rinsing cycles and ethanol treatments, and ultimately dried. As a result, the glands figured via µCT scans should appear empty, which they do in most cases. The residues found in the glands of the aforementioned species are presumably preparation artifacts. Without precise knowledge of the chemical nature of the probably different secretions, it is uncertain whether these substances might have reacted with the Bouin’s fixative, ethanol or iodine. However, this does not affect size and structure of the glands.

Understanding the morphological diversity, or disparity, of defensive glands across the various phasmatodean taxa is not possible without also incorporating knowledge on the chemical nature of the repellent substances. A smaller gland might be more powerful in repelling predators when the chemical repellent is more effective than a bigger gland emitting a less effective substance. In fact, the anatomical diversity of the prothoracic glands is mirrored by the glands’ huge diversity of chemical compounds. To date, at least 27 substances have been reported in twelve species [31, 37]. Several of the known substances have reported repelling effects against predators such as spiders, ants, mosquitoes, beetles, parasitic wasps, mice, rats, frogs, lizards, and birds [15, 20, 35–37]. In various experiments, Thomas Eisner (1965, 1997) [13, 20] demonstrated the repellent secretions’ effectiveness of *Anisomorpha buprestoides* (anisomorphal, a monoterpene) and *Oreophoetes peruana* (quinoline, a heteroaromatic compound) individuals by exposing them to various potential attackers. In the leaf insect *Cryptophyllium westwoodii*, three different pyrazines were identified as major components of the repellent secretion but were not tested for their effectiveness [34]. Nevertheless, pyrazines have been reported to have repelling effects on ants, rats and birds and are also used for defense in monarch butterflies and Zygaenidae moths [52–55]. Therefore, we assume a similar function for *C. westwoodii*. Unfortunately, the chemical compound is not known for *Ph*. *philippinicum*, nor were the defensive glands illustrated for *C. westwoodii* (therein referred to as *Phyllium westwoodii*) by Dossey et al. (2009).

The substances listed appear to have similar effects on attackers, yet they all belong to different substance classes. These compounds strongly differ in their quality and quantity (depending on gland size) and probably are highly specific towards certain predators [56]: For instance, the European wood tiger moth (*Arctia plantaginis*) produces different repellent secretions in its thoracic glands and in the glands of the abdomen [55]. While the secretions stemming from the thorax repel birds, but not ants, the secretions from the abdomen repel ants, but not birds. Due to the general lack of knowledge of phasmatodean ecology, hardly anything is known in regard to specific predators. Since stick and leaf insects inhabit a huge variety of habitats, ranging from the forest ground up to the canopy of tropical rainforests [57], they must be confronted by a huge variety of predators. Thus, the specific predation selection pressures are probably responsible for the observed differences in the repellent glands’ morphology and chemistry [58]. However, when encountering a high predator diversity, it appears disadvantageous to focus solely on the most effective defense against a single predator, but to develop a more generally efficient repellent [55]. This might explain the broad effectiveness of the secretion produced by *A. buprestoides* and *O. peruana*.

Stick and leaf insects usually make use of a combination of various primary and secondary defensive strategies beyond chemical defense or its display via aposematism. Those strategies comprise masquerade and crypsis as the most prominent primary defense and escape via running or flying as a common secondary strategy, but also thanatosis, leg autotomy, defensive stridulation, active counterattack via heavily armed legs, and startle display of legs and/or wings are deployed [6, 27, 59–61]. However, wings are absent in the majority of species [62] not allowing for flight, startle display or defensive stridulation, as is also the case for *A. buprestoides* or *O. peruana* who fully rely on aposematic coloration and chemical defense. It is argued that a particular evolutionary advantage arises from a single adaptation towards a broad-ranged array of attackers is more frequently selected and eventually evolves more quickly [63]. This would allow a species to abandon other defensive strategies, to reduce wings, shift away from a cryptic lifestyle and eventually develop a striking aposematic coloration [2, 64]. However, the interrelation between the listed factors appears to be even more complex, not allowing for simple explanations. For instance, we can neither see any strong relation between the absence of wings or flight ability and gland size nor between masquerade crypsis and gland size. Some slender, twig-imitating taxa in fact rely heavily on camouflage and consequently exhibit extremely small rudimentary prothoracic glands (cf. Figure 9, *Clonopsis gallica*, *Carausius morosus*, *Lobofemora scheirei*). In contrast, the leaf insect *Ph*. *philippinicum* has unexpectedly large glands as outlined above, although leaf insects imitate angiosperm leaves to perfection via lobe-like expansions on body and legs and fore wing veins imitating the pattern of leave venation [65] (Fig. 1G;). However, it might be similar to what has been reported for the tiger moth (see above), that different parts of the defense repertoire of a species might be directed against specific predators, i.e., the leaf mimicry against visually hunting predators such as birds and mammals and the chemical defense against invertebrates such as ants. It is also noteworthy that female leaf insects furthermore perform defensive stridulation with stridulatory files on their antennae, which is interpreted as a defense mechanism against acoustically hunting bats [66].

We did not see any correlation between the presence or absence of wings and gland size either. Species that are capable of flighted escape could be less dependent on chemical defense than less mobile species, yet one of the largest glands is found in *Anarchodes* (Fig. 8A) which has well developed hind wings and is capable of ascending flight (pers. obs.), yet this species exhibits aposematic coloration including startling display by showing its strikingly red hind wing upon disturbance [51].

### Outlook

Stick and leaf insects are notorious for exhibiting a high degree of phenotypic plasticity and homoplasy in evolution, affecting multiple character systems such as wings [62, 67], reproductive strategies and eggs [68, 69], tarsal attachment structures [70, 71] – and the prothoracic defensive glands appear to be no exception in this regard. For understanding the evolution of the prothoracic repellent glands in stick and leaf insects, the evolutionary reconstruction of gland anatomy will become necessary based on a much more extensive and taxonomically denser sampling of the various phasmatodean lineages. The focus on individual subgroups such as Necrosciinae, Phylliidae and Pseudophasmatinae, will clarify whether the taxa chosen in the present study are representative for the respective clades. Furthermore, we will add data on male individuals in order to be able to infer potential sexual dimorphism, and more detailed anatomical investigations of the musculature’s fine architecture will become necessary to understand functional aspects of secretion emittance. At present, the information on the chemical nature and the morphology of defensive glands is sparse and disconnected, i.e., for most species whose repellent substance is known, the gland morphology is unknown, and vice versa. This needs to be augmented for the missing data and will make extensive chemical analyses a crucial next step. In combination with data on lifestyle and additional aspects on anti-predator defense of the taxa in question, a more complete picture on the natural history of this complex and impressive character system will likely emerge.

## Material and methods

### Specimens

The phasmid species used in this study (see Table 2) originated from our lab-cultures at the Department of Animal Evolution and Biodiversity of the University of Göttingen, except for *Timema douglasi*, which we obtained from the research group of Tanja Schwander, University of Lausanne, Switzerland. In this study, we used exclusively adult females. The species examined are all based on breeding cultures that were established before 2014, so consequently are not affected by the Nagoya protocol. *Lobofemora scheirei* was collected in Vietnam through the Capacity Building Programme of the Belgian Global Taxonomic Initiative National Focal Point that is still active and in full agreement with the Convention of Biological Diversity including the Nagoya Protocol of Access and Benefit Sharing.

**Table 2:** Overview of the phasmatodean species examined in this study

Phasmatodea		
Timematodea	*Timema douglasi* Sandoval & Vickery, 1996	California, US
Euphasmatodea		
Aschiphasmatinae	*Orthomeria kangi* Vallotto, Bresseel, Heitzmann & Gottardo, 2016	Philippines
Neophasmatodea		
Occidophasmata		
Pseudophasmatidae	*Pseudophasma subapterum* (Redtenbacher, 1906)	Venezuela
Diapheromerinae	*Oreophoetes peruana* (Saussure, 1868)	Peru
Oriophasmata		
Heteropterygidae	*Tisamenus fratercula* (Rehn & Rehn, 1939)	Philippines
Bacillinae	*Clonopsis gallica* (Charpentier, 1825)	Southwest Europe
Phylliidae	*Phyllium philippinicum* Hennemann, Conle, Gottardo & Bresseel, 2009	Philippines
Lonchodinae	*Carausius morosus* (Brunner von Wattenwyl, 1907)	India
Necrosciinae	*Anarchodes annulipes* (Gray, 1835)	Malaysia
Clitumninae	*Lobofemora scheirei* Bresseel & Constant, 2015	Vietnam
Cladomorphinae	*Taraxippus samarae* Conle, Hennemann & Valero, 2020	Costa Rica
Lanceocercata	*Dimorphodes* sp.	Indonesia

The animals were anesthetized in the refrigerator at 4 °C together with a small tissue paper soaked with 3–5 droplets ethyl acetate and subsequently cut the metathorax at its posterior end, detaching head and thorax from the remaining body. Antennae and legs were cut near the body. The specimens were fixated in 70% Bouin’s solution for 70 h following an ascending EtOH row and 1% iodine staining for 18 h. Critical point drying was done with the BALZER CPD030.

### Imaging and image data processing

For the µCT scans, the specimens were glued vertically on small parts of polystyrene cut to the required size and afterwards stacked in various numbers (depending on the size) in a polyimide tube (10 mm diameter) which lastly was glued on a specimen stub (agar scientific 0.5″). Individual samples were glued on specimen stubs alone.

We used the EasyTom μ-CT system (RX Solutions, France) equipped with a sealed X-ray tube (Hamamatsu L12161-07) with a tungsten (W) target and a spot size down to 5 μm (small focal spot mode). Projection images were acquired with a CCD detector (Gadox-scintillator, 9 × 9 μm^2^ pixel size, 2 × 2 binned). Parameters were varied empirically to suit the respective specimen with tube voltages from 40 to 80 kV and geometric magnifications in the range from 2 to 8, resulting in voxel sizes between 2 µm and 9 µm. Typical values for the number of projections and accumulation times were chosen around 1568 and 3 s, but were adapted according to the contrast, size of the organism and available total scan times, which ranged between 3 and 16 h. For the data shown, Supplementary table 1 gives the exact experimental parameters for each scan. The data was reconstructed using the software provided with the instrument.

Image processing was done using Amira 2021.1. Glands and digestive system were labeled and afterwards progressed with Biomedisa semi-automatic segmentation platform [72]. 3D visualizations were done with volume rendering and surface generating functions and subsequently processed with Affinity Photo 2.0.3 and Affinity Designer 2.0.3.

Living animals were photographed using a Canon EOS90D DSLR camera attached to a camera tripod.

### Gland/lumen volume and prothorax volume measuring

Due to enormous body size differences between species, the glandular volume is set in relation to the prothorax volume to provide a reference value (gland-prothorax ratio) for interspecific comparisons. While meso- and metathorax are often strikingly elongated, the prothorax remains short, even in the most elongated stick-like forms, making the prothorax an ideal reference volume. The prothorax was considered as an elliptical cylinder. As fixed points, we defined eight points on the prothorax (dorsal prothorax midpoint anterior & posterior, ventral prothorax midpoint anterior & posterior, left and right lateral prothorax midpoint anterior & posterior) to determine the dorsal length, ventral length, lateral length (left and right), height (anterior and posterior), width (anterior and posterior) of the cylinder (illustrated in Supplementary Fig. 1) and calculate its volume with the formula V = ra*rb*π*h. The lengths were measured with the line probe tool in Amira. Gland volume and lumen volume were measured with the material statistics tool in Amira 2021.1.

Since the relative amount of musculature varies enormously between glands of different taxa (e.g., Fig. 2C + F), we did not only measure the total gland volume in relation to the prothorax, but also the proportion of the lumen in relation to the whole gland itself (lumen-gland ratio, lgr) to indirectly determine the musculature content.

Repellent gland types in their absolute size were mapped onto the phylogeny of the examined species based on Simon et al. 2019 [7].

### Supplementary Information

**Additional file 1. Supplementary Figure 1.** Defined fixed points on the prothorax for volume measurement: Dorsal prothorax midpoint anterior (d1) & posterior (d2), ventral prothorax midpoint anterior (v1) & posterior (v2), left and right lateral prothorax midpoint anterior (l1, r1) & posterior (l2, r2).

**Additional file 2. Supplementary Table 1.** Overview of the µCT scans measurement data.

## Data Availability

The µCT data sets used in this study can be accessed via: data.goettingen-research-online.de.
