# Supplementary material for: High disparity in repellent gland anatomy across major lineages of stick and leaf insects (Insecta: Phasmatodea)
Source: BMC Zool. 2024 Jan 2;9:1. doi: 10.1186/s40850-023-00189-2 (PMC10759571; doi:10.1186/s40850-023-00189-2)
Supplement: Supplementary file 1 — Additional file 1. Supplementary Figure 1. Defined fixed points on the prothorax for volume measurement: Dorsal prothorax midpoint anterior (d1) & posterior (d2), ventral prothorax midpoint anterior (v1) & posterior (v2), left and right lateral prothorax midpoint anterior (l1, r1) & posterior (l2, r2). [file 40850_2023_189_MOESM1_ESM.docx]

**Supplementary Figure 1**
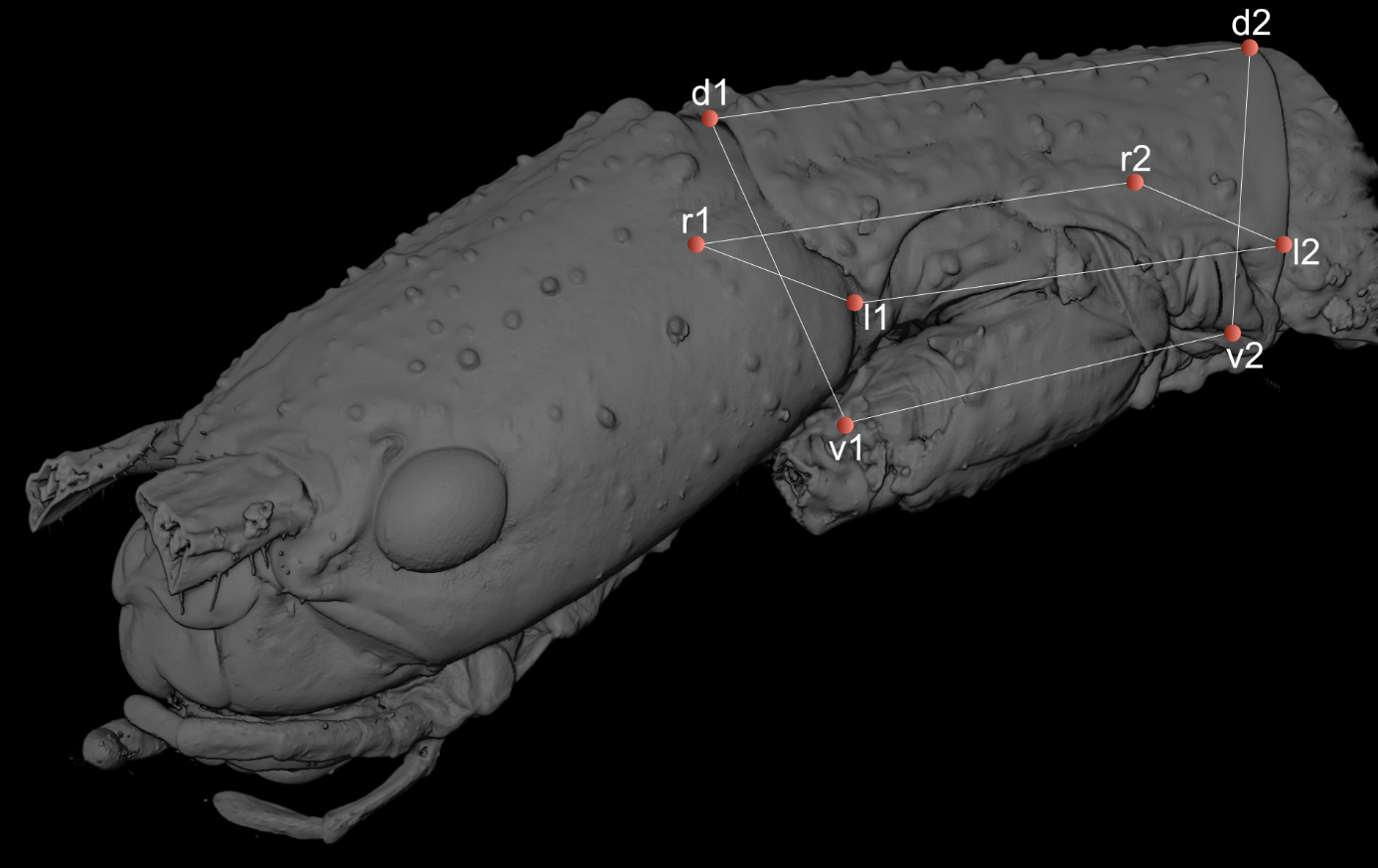


Supplementary Figure 1: Defined fixed points on the prothorax for volume measurement: Dorsal prothorax midpoint anterior (d1) & posterior (d2), ventral prothorax midpoint anterior (v1) & posterior (v2), left and right lateral prothorax midpoint anterior (l1, r1) & posterior (l2, r2).
