# Supplementary material for: High disparity in repellent gland anatomy across major lineages of stick and leaf insects (Insecta: Phasmatodea)
Source: BMC Zool. 2024 Jan 2;9:1. doi: 10.1186/s40850-023-00189-2 (PMC10759571; doi:10.1186/s40850-023-00189-2)
Supplement: Supplementary file 2 — Additional file 2. Supplementary Table 1. Overview of the µCT scans measurement data. [file 40850_2023_189_MOESM2_ESM.docx]

Supplementary Table 1: Overview of the µCT scans measurement data

|  | tube voltage / keV | magnification | voxel size / μm | N_prj | accumulation time / s | ccdimages | ccdexposure / s | total scan time / min | number of turns | overhead in % | det px | Source-object-distance | Source-detector-distance |
| --- | --- | --- | --- | --- | --- | --- | --- | --- | --- | --- | --- | --- | --- |
| *Timema douglasi* | 80 | 5 | 3.53 | 1440 | 6 | 3 | 2 | 190 | 1 | 31.94 | 18.00 | 26.30 | 134.16 |
| *Orthomeria kangi* | 60 | 8 | 2.30 | 1568 | 6 | 3 | 2 | 180 | 1 | 14.80 | 18.00 | 14.64 | 114.73 |
| *Pseudophasma subapterum* | 40 | 3 | 6.72 | 1568 | 3 | 3 | 1 | 210 | 2 | 33.93 | 18.00 | 55.45 | 148.48 |
| *Oreophoetes peruana* | 40 | 4 | 4.54 | 1568 | 24 | 6 | 4 | 660 | 1 | 5.23 | 18.00 | 34.61 | 137.21 |
| *Tisamenus fratercula* | 40 | 3 | 6.28 | 1568 | 3 | 3 | 1 | 210 | 2 | 33.93 | 18.00 | 51.82 | 148.48 |
| *Clonopsis gallica* | 50 | 4 | 4.58 | 1568 | 3 | 3 | 1 | 110 | 1 | 40.31 | 18.00 | 35.89 | 141.10 |
| *Phyllium philippinicum* | 40 | 4 | 4.74 | 1568 | 3 | 3 | 1 | 315 | 3 | 33.93 | 18.00 | 37.79 | 143.39 |
| *Carausius morosus* | 60 | 6 | 3.17 | 1568 | 3 | 3 | 1 | 105 | 1 | 33.93 | 18.00 | 20.70 | 117.42 |
| *Necroscia annulipes* | 50 | 2 | 8.22 | 1568 | 3 | 3 | 1 | 210 | 2 | 33.93 | 18.00 | 77.76 | 170.34 |
| *Lobofemora scheirei* | 50 | 3 | 7.12 | 1568 | 3 | 3 | 1 | 110 | 1 | 40.31 | 18.00 | 62.71 | 158.58 |
| *Taraxippus samarae* | 60 | 3 | 6.52 | 1568 | 32 | 8 | 4 | 940 | 1 | 12.40 | 18.00 | 52.41 | 144.73 |
| *Dimorphodes* sp. | 40 | 3 | 6.28 | 1568 | 3 | 3 | 1 | 210 | 2 | 33.93 | 18.00 | 51.80 | 148.48 |
